# Diabetic Foot: Surgical Approach in Emergency

**DOI:** 10.1155/2013/296169

**Published:** 2013-10-23

**Authors:** C. Setacci, P. Sirignano, G. Mazzitelli, F. Setacci, G. Messina, G. Galzerano, G. de Donato

**Affiliations:** ^1^Vascular and Endovascular Surgery Unit, Department of Medicine, Surgery and Neurological Sciences, University of Siena, Viale Bracci 1, 53100 Siena, Italy; ^2^Area of Public Health, Department of Molecular and Developmental Medicine, University of Siena, Siena, Italy

## Abstract

*Introduction*. Critical limb lschemia (CLI) and particularly diabetic foot (DF) are still considered “Cinderella” in our departments. Anyway, the presence of arterial obstructive disease increases the risk of amputation by itself; when it is associated with foot infection, the risk of amputation is greatly increased. *Methods*. From January 2007 to December 2011, 375 patients with DF infection and CLI have been admitted to our Unit; from 2007 to 2009, 192 patients (Group A) underwent surgical debridement of the lesion followed by a delayed revascularization; from 2010 to 2011, 183 patients (Group B) were treated following a new 4-step protocol: (1) early diagnosis with a 24 h on call DF team; (2) urgent treatment of severe foot infection with an aggressive surgical debridement; (3) early revascularization within 24 hours; (4) definitive treatment: wound healing, reconstructive surgery, and orthesis. We reported rates of mortality, major amputation, and foot healing at 6 months of followup. *Results*. The majority of patients in both groups were male; no statistical differences in medical history and clinical condition were reported at the baseline. The main difference between the two groups was the mean time from debridement to revascularization (3 days in Group A and 24 hours in Group B). After 6 months of follow-up, mortality was 11% in Group A versus 4.4% in Group B. Major amputation rate was 39.6% and 24.6% in Groups A and B, respectively. Wound healing was achieved in 17.8% in Group A and 20.8% in Group B. *Conclusions*. This protocol requires a lot of professional skills that should to reach the goal to avoid major amputations in patients with DF. Only an interdisciplinary integrated DF team and an early intervention may significantly impact the outcome of our patients: “Time is Tissue”!

## 1. Introduction

Diabetes is a chronic disease that approximately involves 350 million people (6.5%) worldwide, with an increasing trend to some 440 million (7.8%) by 2030 [1]. It is burdened by microangiopathic (nephropathy, retinopathy, and neuropathy) and macroangiopathic complications (cardiovascular disease and fatal or nonfatal stroke). 

Cardiovascular diseases are the leading cause of morbidity and mortality in diabetes mellitus, especially in type II [2]. Overall, myocardial infarction, fatal or nonfatal stroke, and amputations are 2 to 4 times more frequent, and global cardiovascular risk is about 3 times higher in diabetic patients than in the nondiabetic population [3].

By the way, in a Finnish study, mortality in patients with type II diabetes without prior myocardial infarction turned out to be even equal to that of nondiabetic subjects with prior myocardial infarction [4].

Peripheral arterial disease (PAD) represents a continuum of disease entities that range between asymptomatic PAD, stable symptomatic intermittent claudication, CLI, acute limb ischemia, and amputation. CLI is defined as PAD causing resting lower-extremity pain at rest and having threatened or frank tissue loss and is classified as Rutherford-Becker Classes 4–6 or Fontaine Classes III and IV. CLI is a disabling disease and represents the end of the spectrum of PAD prior to tissue and limb loss. Other definitions for CLI have been suggested to include absolute pressures of ankle pressure <50–70 mm Hg, toe pressure <30–50 mm Hg, or reduced TCPO_2_ <30–50 mm Hg.

Diabetic foot (DF) is one of the main complications of diabetes mellitus; it involves approximately 15% of diabetic patients [5, 6] and represents the main cause of amputation in developed countries [7–9]. DF is a multifactorial disease, since neuropathy, peripheral vasculopathy, and a lower resistance to infections contribute to its development [10].

Feet complications, closely related to neuropathy and obstructive peripheral vascular disease, are responsible for more than 1 million leg amputations every year [11].

The presence of foot infections could dramatically improve the risk of amputation. Frequently, DF complicates the clinical course of ulcerative lesions of the foot and also greatly increases the risk of amputation, especially when associated with a severe deficiency of blood tissue perfusion [12].

The treatment of DF ulcers significantly depends on the vascularization and the presence of infectious process.

Treating an infected lesion without having secured an appropriate vascular support does not make sense. The foot needs greater vascular support to heal than what is needed not to get sick.

Anyway, the presence of arterial obstructive disease increases the risk of amputation by itself; when it is associated with foot infection, which frequently complicates the clinical course of DF, the risk of amputation is greatly increased [13–15].

A main issue is who and when to treat a patient with DF. No consensus is reported in the literature about health-care professionals involved in these fields and about the timing of the treatments.

The aim of this work is to analyse whether the introduction of a new multidisciplinary protocol might change the outcome of our patients in terms of mortality, morbidity, major amputation, and wounds healing.

## 2. Materials and Methods

From January 2007 to December 2011, 375 patients with DF infections and CLI have been admitted to our center of Vascular and Endovascular Surgery.

The treatment of these patients has always been characterized by interdisciplinarity with implication of various professionals in the several stages of the long process of healing.

Since January 2010 our center adopted a new-shared protocol that was applied to all treated patients.

The protocol is divided into four phases and provides the following:

1. early diagnosis with a 24 h on call DF team. All the members of the team should be able to perform a duplex scan and to identify an infective disease, if present;
2. urgent treatment of severe foot infection with an aggressive surgical debridement;
3. early revascularization within 24 hours. In all cases the first line approach should be represented by endovascular procedures (PTA ± stenting);
4. definitive treatment: wound healing, reconstructive surgery, and orthesis.

On the basis of these protocols, we divided our experiences into two different phases: from 2007 to 2009, 192 patients (Group A) underwent surgical debridement of the lesion followed by a delayed revascularization; from 2010 to 2011, 183 patients (Group B) were treated following the described protocol. Demographic, clinical, and intraoperative variables were entered into a specific database by the operating team. Data were collected in a computerised database and were analysed prospectively.

### 2.1. Surgical Treatment

All patients underwent clinical examination, ABI (ankle-brachial index) measurement, and ultrasound examination before treatment. The angiography was performed at the same time as the procedure in order to map the femoropopliteal lesions accurately and thus optimize the revascularization strategy. All patients were treated by a vascular surgeon in an operating theatre equipped with a portable fluoroscopy unit (GE-OEC 9800; GE Medical Systems, Salt Lake City, UT, USA).

In our center anterograde ipsilateral percutaneous femoral access was preferred when at least 5 cm of a patent proximal segment of superficial femoral artery (SFA) was evident at ultrasonography. A contralateral approach via a cross-over long sheath was only used in the presence of either SFA occlusion in its origin, high femoral bifurcation (documented by ultrasound), or obesity. We were able to check the correct localisation of the common femoral artery puncture and reduce the risk of retroperitoneal bleeding using micropuncture sets and contrast injections under fluoroscopy.

A soft, angled, hydrophilic 0.035′′ guidewire in combination with a 5-F, angled, hydrophilic catheter was brought near the origin of the occlusion.

Advancing the guidewire through the true lumen was attempted in all cases. When needed, the subintimal plane was entered by forming a loop at the end of the guidewire and advancing it, along with the catheter, across the occluded arterial segment. A reentry device was used (Outback, Cordis Corporation, Miami Lakes, FL, USA, in all cases) only when recanalisation by simple subintimal angioplasty (SAP) was unsuccessful. Following confirmation of catheter reentry into the true lumen, balloon angioplasty was used to dilate the subintimal channel. Stenting was performed only when residual stenosis was >30% or there was a flow-limiting dissection.

We tried to use a standardised approach: a brief SAP procedure of 30–40 min and use of a reentry device are advised when accessing the true lumen proves difficult, so as not to dissect the popliteal artery or threaten the supragenicular collaterals. If the procedure cannot be concluded safely, we continued the intervention surgically or used a hybrid approach. The presence of a vascular surgeon in the team is important, as in the case of a failed SAP, the first intervention should not preclude the possibility of further surgical revascularization [16].

Our surgical or endovascular approach is oriented to respect the angiosome concept due to the necessity of a direct blood flow to the wound related arteries [17].

### 2.2. Statistical Analysis

The Kaplan Meier method was used to show the trend in the two groups. The log rank test was used in order to detect if there were any statistically significant differences between the two curves. Significance level was set, (*P* < 0.05); Stata SE, version 12.1, StataCorp, College Station, Texas, USA software was used for the analysis.

We report mortality rate, major amputation rate (defined as above the ankle amputation), and wound healing rate in both groups at 6 months of followup. Minor amputation below the ankle was considered as a wound healing when function of the limbs was conserved, and as nonhealing ulcer in other cases.

Limb function in below the ankle amputation was conserved by the use of appropriate orthesis that ensures the discharge of the affected area in the early postoperative period. These devices allow achieving a weight bearing of the affected area from amputation (whether calcaneal tarsal or metatarsal) by sole rocking. After successful healing, it is possible to prescribe custom-made shoes with rigid filling to ensure proper mobility.

## 3. Results

The majority of patients were male in both groups. No significant differences in terms of age or comorbidities were recorded in the present series. Demographic characteristics of patients in both groups are described in Table 1. All patients were treated in urgent or emergent settings.

The main time between debridement and revascularization was 3 days (range 1–7 days) in Group A; all patients in Group B were revascularized within 24 h from the surgical debridement. In our experience, even in very complex cases, a primary amputation was never performed.

As described above, all patients underwent first stage endovascular procedure. Only in case of endovascular failure, an intraoperative surgical conversion was performed. Endovascular revascularization was successfully performed in 84.7% of the patients. Stenting was performed only in a bail-out situation. Open surgical conversion was performed intraoperatively in all cases of failure in endovascular recanalization.

In all cases, the patients executed a specific antibiotic therapy based on a previously performed antibiogram.

**Figure 1:**
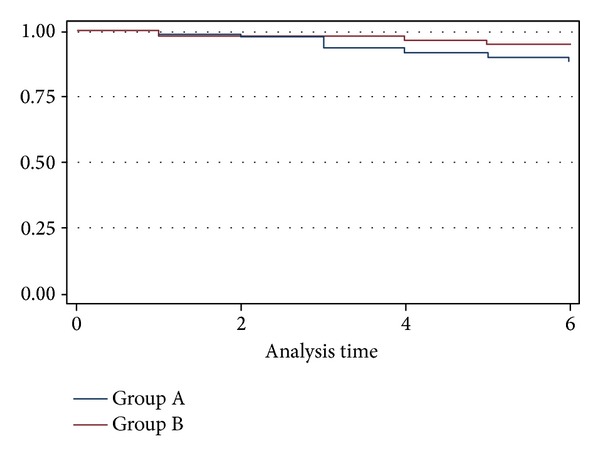
KM estimates survival rates.

At six months of followup we report 22 (11%) deaths in Group A and 9 (4.4%) deaths in Group B, which represents a statistically significant difference between the two groups (*P* = 0.0224 and HR = 0.41) (Figure 1).

In Group A we reported 2 deaths (1.04%) due to septic shock; both patients were septic at moment of clinical presentation. An endovascular recanalization with restoration of direct flow to the foot was achieved in both cases. Unfortunately, both patients developed an acute renal failure and a multiorgan failure.

During the followups 12 cases of fatal MIs were observed (6.25%), 5 fatal strokes (2.60%), and 3 renal failures (1.56%).

No case of septic shock was recorded in Group B. Fatal MI was observed in 6 patients during the followup (3.27%), stroke in 2 patients (1.09%), and 1 patient (0.54%) death of colon cancer at 3 months of followup.

**Figure 2:**
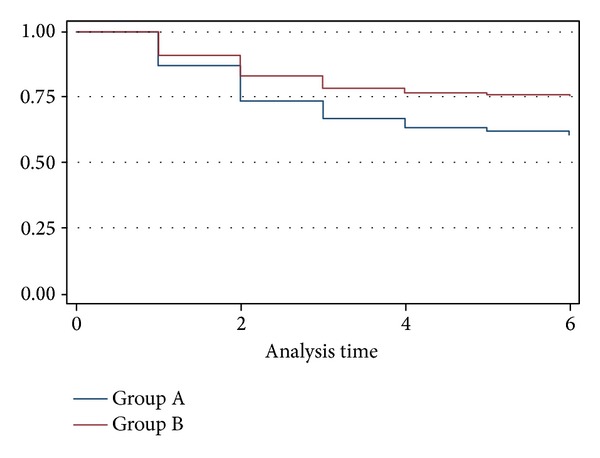
KM estimates amputation rates.

**Figure 3:**
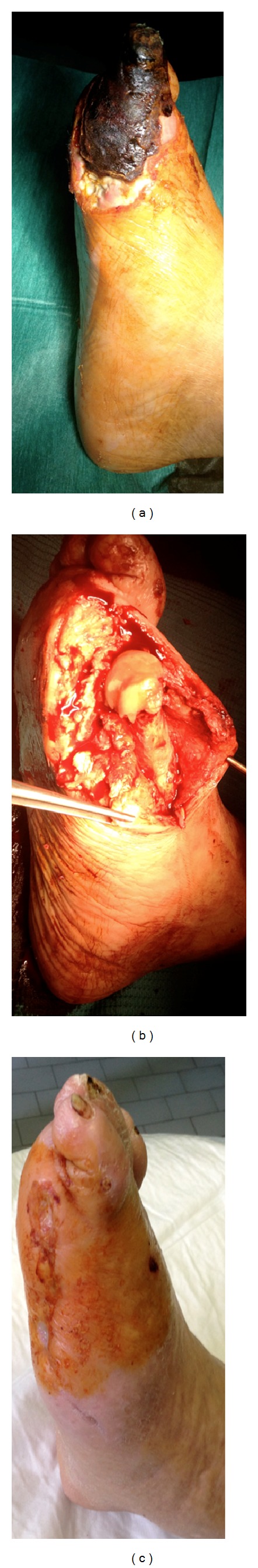
Preoperative (a), intraoperative (b), and follow-up images (c) of DF ulcers.

**Figure 4:**
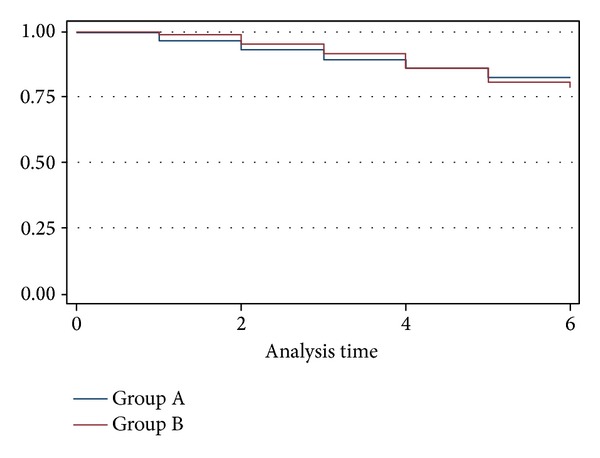
KM estimates wound healing rates.

Major amputation rate was, respectively, 39.6% and 24.6% in Group A and in Group B (*P* = 0.0024, HR = 0.58) (Figure 2). During the follow-up period, all patients in both groups were continuously treated and assisted by vascular surgeons, vascular nurses, and all the other care-providers involved in the healing process by clinical evaluation and wound care. Wound healing (Figure 3) was achieved in 34 patients (17.8%) in Group A and in 39 patients (20.8%) in Group B (*P* = 0.45, HR 1.18%) (Figure 4).

## 4. Discussion

Critical limb ischemia and in particular DF are still considered “Cinderellas” in our departments. And this is difficult to explain if we consider that every year more than one million people suffer from a lower limb amputation as a result of diabetes. It is hard to believe that although 85% of all amputations are preceded by the development of foot ulcers, the prevalence of amputations ranges oscillates from 0.2 up to 4.8% [18].

We must consider that the complications associated with the diabetic disease are difficult to manage and require a significant commitment in terms of health care [19, 20].

Prompers et al. reported that the presence of critical limb ischemia greatly increases the risk of major amputation. Of note, in their experience, the presence of diabetic neurophathy (even motor or sensory) is linked only to a higher incidence of ulceration; no major risk of amputation was detected [21].

Ulcers, depending on pathogenetical features, can be defined as neuropathic, ischemic, or neuroischemic; all of them can be complicate by a superinfection [22].

Even if many different staging systems of ulcerative lesions have been defined in recent years, the Wagner's classification continues to be the most widely accepted.

Wagner's classification identifies six categories of lesions progressively worsening from stage 0 to stage 5, depending on involvement of different tissue's layers, topographic location, and presence of any infection [23]. This classification allows a clinical diagnosis of the lesion, but on the other side it does not consider the local vascular conditions. This is the main limit because ischemia is the main factor conditioning the clinical evolution of lesions and also the choice between different kinds of treatments [24]. In order to obviate this problem, a new classification, the Texas Wound Classification, which considers also the possible presence of ischemia has been validated.

The Texas Wound Classification demonstrated a positive correlation between dimensions of ulcer, ischemia, and infection with the increase of relative risk of amputation (Table 2). Moreover, it demonstrated a significant positive relationship between the extension and the depth of the infection and the risk of amputation [25]. Early control of infective process represents the main therapeutic goal of emergency surgery in infected DF. The general impression, although not supported by specific prospective studies, is that patients with rest pain and trophic lesions have a worse prognosis than those with only pain and that the greater size of the ulcer worsens the prognosis but only with respect to limb salvage and not for the purpose of patients survival [26].

Diabetes is the most important risk factor for critical limb ischemia [19, 27–30], and it is well recognized that diabetic patients have a high risk rate of both amputation and death compared with nondiabetics [31].

Despite the benefits of pharmacologic therapy, arterial revascularization remains a mainstay in the management of CLI because the restoration of adequate blood flow to the foot is crucial to provide pain relief, promote wound healing, and avoid amputation. Although surgical revascularization is an important therapeutic option, recent data supports the use of percutaneous transluminal angioplasty, which is both feasible and safe in this setting [32–35]. However, recent reports from the literature seem to suggest the positive role of new commercially available drugs in order to prevent ulceration in diabetic patients. These findings might completely change the scenario in the future [36].

More aggressive techniques have been developed to improve the results of percutaneous transluminal angioplasty in vessels below the knee. Techniques such as subintimal angioplasty [37], retrograde approach with transpedal access [38], subintimal arterial flossing with antegrade-retrograde intervention [39, 40], transcollateral angioplasty [41], and pedal-plantar loop [42, 43] are improving the success rates of percutaneous transluminal angioplasty even in the most distal vascular territories.

By the way, control of any local sepsis through appropriate use of surgical (debridement, drainage, and even amputation) and medical (antibiotics) modalities is always the immediate priority in DF management [44].

Consequently, timing has a key role for the diabetic's foot treatment, especially if it is infected.

Faglia et al. [45] have confirmed how, in case of CLI (especially if it is associated with a severe infection), an early surgical treatment of the infection, followed by early revascularization procedure, can achieve limb salvage or a more distal level of foot amputation. Caravaggi [11] has proposed an “Integrated Surgical Approach” that considers the main aspects of treatment of severe foot infection: time, emergency surgical treatment, and revascularization procedures. Since early surgical treatment of infection is closely correlated with limb salvage, they have underlined that surgical debridement has to be performed as soon as possible regardless of vascular condition of the foot. Revascularization procedures, both surgical or endovascular, are secondary in comparison to the local and systemic infections control.

In conclusion, the need for a coordinate, multidisciplinary care has long been obvious. The recent growth of dedicated amputation prevention centers represents a positive trend, and new drugs could modify the natural history of the disease.

At the moment, we would like to suggest a four-step approach to patients with DF.

1. Early diagnosis with a 24 h on call DF team. All the members of the team should be able to perform a duplex scan and to identify an infective disease, if present.
2. Urgent treatment of severe foot infection with an aggressive surgical debridement.
3. Early revascularization within 24 hours. In all cases the first line approach should be represented by endovascular procedures (PTA ± stenting).
4. Definitive treatment: wound healing, reconstructive surgery, and orthesis.

This solution is also recommended by the most recent guidelines, in particular by International Guidelines on the treatment of diabetic foot and the Guidelines of the European Society of Vascular and Endovascular Surgery of critical limb ischemia and diabetic foot [46].

In our experience many different professional skills should work together 24 h–365 d to reach the goal to avoid major amputations in patients with DF. It is a hard and complex work, but it is proven that only an interdisciplinary integrated diabetic foot Team may lead to a significant impact on the outcome of our patients: “Time is Tissue”!

## Figures and Tables

**Table 1:** Demographic characteristics of the two study groups.

	Group A	Group B
Mean age	75.6	76.7
Male	81.7%	78.6%
Coronary artery disease	63%	64.4%
COPD	35.9%	38.7%
Renal failure	57.8%	58.4%
Hypertension	88.5%	91.8%
Dyslipidemia	75.5%	78.6%

**Table 2:** Texas Wound Classification.

Stage	Grade			
	0	I	II	III
A (no infection or ischemia)	Pre- or postulcerative lesion completely epithelialized	Superficial wound not involving tendon, capsule, or bone	Wound penetrating to tendon or capsule	Wound penetrating to bone or joint
B	Infection	Infection	Infection	Infection
C	Ischemia	Ischemia	Ischemia	Ischemia
D	Infection and ischemia	Infection and ischemia	Infection and ischemia	Infection and ischemia
